# The road not taken: selective sampling and persisting inaccurate impressions

**DOI:** 10.1007/s11031-026-10205-w

**Published:** 2026-02-09

**Authors:** Emily Ann Vanlooy, Chris Harris, Irene van de Vijver, Ruud Custers

**Affiliations:** Utrecht University, Heidelberglaan 1, 3584 CS Utrecht, The Netherlands

**Keywords:** Exploration–exploitation trade-off, Impression formation, Rewarding and aversive outcomes

## Abstract

When individuals learn through experience, they tend to choose those options they believe will produce the most favourable outcomes. Research by Harris et al. (J Exp Psychol: General 149(10):1855–1877, 2020) indicated that this inclination to consistently engage with the perceived optimal choice can bias the resulting evidence, potentially perpetuating inaccurate beliefs under certain conditions. It remains unclear, however, whether this bias persistence depends on the valence of the learning context. In the present study, participants completed a two-armed bandit task with identical options, where initial evidence was manipulated to induce the belief that one option was better than the other. Experiment 1 was conducted in a rewarding context, where financial gains served as a reinforcer (as opposed to neutral outcomes), while Experiment 2 featured a negative context, with the omission of an expected aversive event as a reinforcer. Behavioral measures showed signs of a lasting bias in the aversive context of Experiment 2, corroborated by a bias in explicit contingency estimates. No such effects were observed in the positive context of Experiment 1. These findings suggest that outcome valence modulates bias persistence in experiential learning. Implications for how negative contexts may promote the maintenance of irrational aversive beliefs are discussed.

## Introduction

Choices are inherently plural. Selecting one course of action necessitates the simultaneous dismissal of an array of alternate possibilities. This implements a natural boundary to what we can learn from our experiences, as the unrealized consequences of unselected alternatives remain undiscovered. Additionally, we impose our own limits on learning through expectations and prior beliefs, which guide decisions toward some options and away from others. This leads to a selection bias that distorts the information we access and may perpetuate inaccurate impressions. For instance, at a social event, you might choose to talk to individuals who appear approachable—perhaps because they look familiar or have a friendly face. Whereas this interaction allows you to learn more about them, it limits your opportunity to engage with others. In this way, a pleasant experience may reinforce your preference for interacting with familiar or friendly-looking people, while leaving potential negative assumptions about others unchallenged. Insofar that certain social categories are disproportionately favored in judgments and decisions, this dynamic contributes to the maintenance of stereotypes and discrimination.

That raises the question: what drives these choices, and does it matter? In a social setting, you might be concerned with, for example, having a pleasant conversation and connecting with someone, or you might be focused on avoiding embarrassment and alienation. As these two motives reflect fundamentally different needs, they may affect both sampling strategies, and the way in which outcomes are processed (cf. Regulatory focus theory; Higgins, 1998; Absence-presence distinction; Elliot et al., 2024). Previous studies have demonstrated that adaptive decision-making in a predominantly rewarding environment can lead to persisting biased judgments when the alternative outcome was negative (Bai et al., 2022; Harris et al., 2020; Rich & Gureckis, 2018). However, the learning literature emphasizes the importance of avoiding aversive events for sustaining inaccurate impressions, independent of potential rewards (Lovibond et al., 2009; Pittig et al., 2018; Treanor & Barry, 2017; Van Uijen et al., 2018; Vervliet et al., 2017). In light of both the similarities and disparities between these mechanisms, this paper intends to explore whether the self-perpetuating interplay between selective sampling and biased impressions is similarly evident in positive and negative environments when the alternative outcome is neutral. Specifically, we examine whether biases in decision-making and belief updating emerge in both rewarding and aversive learning environments.

## Sampling as reward pursuit

### Learning from experience: a conflict of interest

The environment does not naturally reveal the outcome distributions of its affordances (cf. Hogarth et al., 2015), meaning that information about the consequences of a specific action is only revealed when the action is executed. As a result, when a choice is made, the opportunity to learn about the outcomes of unchosen alternatives is lost, imposing a limit on the knowledge that can be acquired (Denrell, 2005; Denrell & Le Mens, 2011; Fazio et al., 2004). After a positive social interaction, you cannot know whether the interactions you missed with seemingly less approachable individuals would have been equally pleasant. In the absence of feedback about those foregone interactions, your initial negative assumptions remain untested and unchallenged by experience.

Evidently, an individual’s decision-making process could be influenced by various motives, shaping the way they engage with the information and options presented to them. For example, they could be driven to test the validity of their initial assumptions by engaging with a person of whom they hold a negative impression, allowing them to correct potential inaccuracies. Indeed, some decisions are epistemically motivated—driven by the search for knowledge even at the expense of potential opportunity costs (e.g., Biella & Hütter, 2024).^1^ We argue, however, that epistemic motives are often secondary to hedonic ones. That is, people engage in learning primarily when it is relevant (Eitam & Higgins, 2010) to them and helps them achieve a superordinate goal, such as seeking positive experiences or avoiding negative ones, as stipulated by the hedonic principle (Higgins, 1998; Thorndike, 1927). Consequently, they do not randomly sample the information they consume. Instead, decisions are based on expectations, which are shaped by beliefs or past experiences. This introduces a sampling bias, as decision-makers are more likely to engage with an option of which they hold a favorable impression, compared to an alternative with a relatively low attribute value.

In the context of repeated choices, this implies that choices at any given point in time depend on previous decisions and their outcome. Two key patterns emerge. First, frequent positive interactions with an initial choice can encourage repeated engagement and result in an early (and overly strong) focus on this supposedly superior option (Harris et al., 2020, 2023; Kasper et al., 2023). Secondly, initial negative events foster avoidance. This impedes the correction of negative misconceptions, as the learning process pertaining to that option is halted (cf. “hot stove effect”; Denrell, 2005; Denrell & le Mens, 2011; Denrell & March, 2001; Fazio et al., 2004).

Because both processes influence the exploration–exploitation trade-off inherent to repeated decisions—where people must choose between exploring new alternatives or exploiting a preferred option (Cohen et al., 2007; Hills et al., 2015; Mehlhorn et al., 2015)—they result in a lack of information about those options that are deemed less beneficial, reinforcing early preferences (De Houwer & Hughes, 2020; Harris et al., 2020; Harris et al., 2023; Kasper et al., 2023). Moreover, the drive to explore is not only shaped by hedonic factors but also by the informational value of exploratory choices (Mehlhorn et al., 2015). When the base rate of reinforcement is high, the marginal information gain from exploring alternative options is low, reducing the incentive to explore. In contrast, in environments with lower base rates, exploratory choices yield relatively more informative feedback, making exploration more attractive (Cogliati Dezza et al., 2017). This asymmetry can further amplify early choice biases and contribute to their persistence over time. Even when the sampling bias is not absolute and belief-incongruent evidence emerges occasionally, this is usually insufficient to eliminate biases, especially in ambiguous contexts where some unpredictability is expected (Cohen et al., 2007; Mehlhorn et al., 2015). As such, biased sampling provides an explanatory framework for the persistence of biases: action selection is more likely to be skewed in environments containing frequent reinforcements, whereas infrequent reinforcement may result in a more representative sample of action-outcome contingencies (Harris et al., 2020; Mehlhorn et al., 2015; Teodorescu & Erev, 2014a, 2014b).

### Previous research

The influence of the reinforcement structure of the environment on the persistence of inaccurate impressions was demonstrated by Harris et al. (2020). In this study, participants engaged in a two-armed bandit task, drawing repeatedly with replacement between two choice alternatives resulting in earned or lost points. While the two choice alternatives were identical, one option was more frequently presented in the initial learning phase to induce an illusory correlation between actions and outcomes (cf. pseudocontingencies; Bott & Meiser, 2020; Fiedler et al., 2009; Harris et al., 2020; Meiser et al., 2018). In an infrequent reinforcement condition (reward-impoverished with *p*(+) = .25 and *p*(−) = .75), participants sampled relatively evenly and corrected their initial bias so that they no longer perceived an illusory correlation. In contrast, participants who encountered frequent reinforcement (reward-rich with *p*(+) = .75 and *p*(−) = .25) tended to engage in premature exploitation, leading to bias persistence over the entirety of the task. This effect has been consistently replicated (cf. Harris et al., 2023) and is not confined to reward-seeking behavior. That is, similar effects have been observed in scenarios where participants were encouraged to ‘optimize’ their learning strategies (Kasper et al., 2023). Moreover, the occurrence of this learning trap (cf., Li et al., 2021) does not depend on the specific mechanism through which a bias arises; it can occur whenever exploration involves some implicit cost (Bai et al., 2022).

## Learning in the absence of rewards

Although previous research and theoretical accounts predominantly focused on reward maximization, not every decision-making context contains positive incentives. Biases may still persist through attempts to minimize expected losses or other negative outcomes, as summarized by the hot stove effect (cf. Denrell, 2005). For instance, people may have to choose between the horrible coffee from the department machine, or the somewhat less awful coffee from the supermarket down the road. Furthermore, the finding that negative information transfers more readily than positive information to other stimuli with similar features suggests that inaccurate expectations of aversiveness may be more prevalent in the natural environment than reward-based biases (Fazio et al., 2004). This similarity-based generalization is especially relevant in the context of minority group discrimination, as these groups are typically categorized by salient external features or remarkable mannerisms. Therefore, it is imperative to investigate whether reinforcement learning also facilitates bias perpetuation when expectations are purely negative.

Whereas most of the above-mentioned studies emphasized reward pursuit as the motive underlying biased sampling, the experiments were incentivized through gains and losses simultaneously (cf. Harris et al., 2020, 2023; Kasper et al., 2023; Li et al., 2021). This is not unwarranted, as the theoretical framework surrounding sampling biases does not explicitly mention the nature of reinforcement. The predicted effect relies on two crucial assumptions: (i) information provision must be contingent upon the decision to select one option and forgo its alternative and (ii) that decision must be determined by expectations about the valence of that option relative to alternatives (Denrell, 2005; Fazio et al., 2004; Le Mens & Denrell, 2011). From a behavioral perspective, the choice for one option automatically implies avoidance of the alternatives, so that only relative valence matters for action selection and bias persistence. According to this framework, similar patterns of biased sampling should emerge in exclusively rewarding and exclusively aversive environments..

Nevertheless, it is essential to exercise caution. In a rewarding context, behavior is reinforced if it leads to the unexpected administration of a reward. Conversely, reinforcement in a negative context occurs through the unexpected omission of an aversive event, akin to extinction learning (De Houwer & Hughes, 2020; Vervliet et al., 2013). Given the distinct implications regarding the presence or absence of a stimulus for cognitive functions (Elliot et al., 2024), these differential outcomes do not necessarily have an identical impact on belief-updating. Of particular interest in this framework is the extent to which outcomes are attributed to specific responses, rather than to base rates in the environment. Exploitation only contributes to biased beliefs insofar that the individual assumes these outcomes would not be obtained by choosing the alternative. Extinction, however, is more context-dependent than acquisition, and the absence of an expected outcome is easily attributed to situational factors or concurrent behavior. Such alternative explanations can shield inaccurate prior beliefs from revision (Lovibond et al., 2009; Vervliet et al., 2013).

This suggests that successful avoidance of an aversive event would be more strongly perceived as dependent on the executed response than fruitful reward pursuit. Similarly, a difference in the overall valence of the decision-making context may induce a shift in regulatory focus, potentially resulting in differences in performance, independent of the hedonic principle in itself (Higgins, 1998). As a result, it is uncertain whether the dynamics observed in mixed gain–loss contexts as in Harris et al. (2020) generalize univocally to purely positive or purely aversive contexts. Moreover, it remains unclear whether previously observed effects primarily reflect reward pursuit or avoidance of negative outcomes. The present research aims to address these matters by investigating how learning from experience contributes to bias persistence in environments that are either exclusively rewarding or exclusively aversive.

## The present research

The present research aimed to investigate how the reinforcement structure of the environment affects bias persistence in both positive and negative contexts. Adopting the paradigm from Harris et al. (2020), we conducted an experiment with positive incentives (Experiment 1) and one with negative incentives (Experiment 2). Critically, in both experiments the alternative outcome was always neutral, rendering this outcome either relatively negative or relatively positive. Each experiment contained a frequent reinforcement (FR) condition, where relatively speaking, positive outcomes were frequent. In Experiment 1, positive outcomes were contrasted with (relatively negative) neutral outcomes, whereas in Experiment 2, (relatively positive) neutral outcomes were contrasted with negative outcomes. These conditions are consistent with the findings that behavior can be reinforced by the omission of an expected aversive stimulus, triggering similar neural activity as receiving a reward does (Erdeniz & Done, 2019; Kim et al., 2006; Vervliet et al., 2017; Wrase et al., 2007).

Both experiments served to investigate three hypotheses. First, we hypothesized that an uneven presentation of evidence would induce an initial bias through a pseudocontingency mechanism (cf. Bott & Meiser, 2020; Eder et al., 2011; Fiedler et al., 2009), so that participants would associate the most frequently shown option with the most prevalent outcome. Although a deviation from this first hypothesis would not threaten the interpretability of our main findings, we included this hypothesis as a manipulation check to ensure that participants were indeed biased on average before starting information sampling. Additionally, we expected the reinforcement structure of the environment to affect the exploration–exploitation trade-off. Frequent reinforcements (FR) were predicted to promote premature exploitation, whereas infrequent reinforcements (IR) were expected to encourage greater exploration. Thirdly, the differential impact on sampling behavior was anticipated to lead to bias perpetuation in the FR condition and bias correction in the IR group.

While these hypotheses are nearly identical to those formulated by Harris et al. (2020), the current study differed considerably. As mentioned above, both experiments contained a neutral outcome, to allow a clear distinction between reinforcement learning based on positive and negative incentives. This way, we aimed to establish the relevance of distinguishing between those types of learning, and to explore the importance of avoidance behavior in bias persistence. Besides the implications for social psychological research, this could potentially extend the application of sampling paradigms to the field of clinical fear and avoidance.

## Experiment 1

### Methods

The purpose of Experiment 1 was to replicate the findings reported by Harris et al. (2020) within a context devoid of losses. This facilitates the examination of whether the pursuit of rewards in a primarily rewarding environment alone is sufficient to elicit premature exploitation of an initial bias and impede belief updating.^2^

The present research adheres to the ethical guidelines established by the Ethics Review Board of the Faculty of Social and Behavioral Sciences at Utrecht University (19-155). The studies were conducted in English via Soscisurvey (Leiner, 2021). Participant recruitment, hypotheses, experimental design and data analyses were preregistered before the start of data collection (see https://aspredicted.org/Q9K_7RZ).

#### Participants

An a priori power analysis was performed using the “pwr” package in R (Champely, 2020) to calculate the recommended sample size for a one-sided *t*-test of two independent samples. The input parameters included the effect size for the difference in ∆P (*d* = 0.29) reported by Harris et al. (2020), a power of *(1–β)* = .80 and a significance level *α* = .05. The analysis yielded a recommended sample size of 148 participants per condition, resulting in a total of *N* = 296.

Participants were recruited via Prolific Academic (https://prolific.co/), with inclusion criteria stipulating fluency in English, a minimum approval rating of 95 out of 100 on the platform and residency in the US, UK, Canada, Australia, New Zealand or a European country with similar living standards (see Appendix A for the full list of included countries). While we acknowledge that the latter criterion limits the diversity of our sample, it was included to avoid excessive discrepancies in the valuation of the monetary incentives used in the experiments. Participants who failed more than one out of three attention checks were excluded from the dataset. The final sample comprised *N* = 300 participants (${N}_{female}$= 153) with an average age of 33.17 years (*SD* = 11.70), who received a minimum financial reward of £1.5 for their participation. Depending on their performance in the decision-making task, participants could earn up to £1 in bonus payments, with an average of £0.49.

#### Design

The study implemented a between-subjects design with the base rate of outcomes as the independent variable, which was manipulated to yield either frequent or infrequent rewards. In a two-armed bandit task, participants were asked to make a series of consecutive decisions, each involving choosing between two bags, labelled ‘A’ and ‘B’, and thereby drawing a ball from the chosen bag. The bags contained blue and yellow balls, with a predetermined ratio that was unknown to participants. The outcome probability for both bags was identical, as the ratio of blue to yellow balls was either 3:1 or 1:3 for both bags. Participants were randomly assigned to a frequently reinforcing or an infrequently reinforcing decision-making environment. In the former, drawing the frequent ball resulted in a gain of 10 points, while the infrequent ball led to no gain. Conversely, in the latter, the infrequent ball resulted in the gain of 10 points, while the frequent ball led to no gain. Therefore, the base rate probability of gaining a positive outcome was 75% in the FR condition and 25% in the IR condition. As such, the study had a two-factor between-participants design, where the frequency of colors of the balls and the direction of questions regarding conditional probability estimates were counterbalanced within conditions (see also Table 1 for an overview over the two conditions).

**Table 1:** Overview over conditions for both experiments

	Experiment 1		Experiment 2	
	Frequent ball (e.g., blue)	Infrequent ball (e.g., yellow)	Frequent ball (e.g., blue)	Infrequent ball (e.g., yellow)
	75%	25%	75%	25%
Frequent reinforcement condition (FR)	Win	Neutral	Neutral	Loss
Infrequent reinforcement condition (IR)	Neutral	Win	Loss	Neutral

Both choice alternatives (bags) contained the exact same number of balls. The distribution of balls and their respective outcomes varied per condition as summarized here

#### Procedure

Following active consent, the experiment was divided into three phases: an initial task, a learning phase, and a free-sampling phase.

##### Initial task

The initial task was included in this study to make it consistent with Experiment 2, in which the task was used to endow people with money, so that losses could be used as incentives in the primary task. Therefore, further elaboration on the initial task is provided in the Methods section of Experiment 2. Regardless of their performance, each participant received the feedback that they had earned £0.20 with this task. Subsequently, they proceeded to complete a demographics questionnaire consisting of an attention check and questions pertaining to their age, gender, country of residence and intentions for the earnings they received from the experiment.

##### Learning phase

Following the demographics questionnaire, participants proceeded to the main part of this study: the decision-making task. This was the two-armed bandit used by Harris et al. (2020) that was previously introduced. To reiterate, participants sampled from two bags labelled ‘A’ and ‘B’, which both contained the same proportion of blue and yellow balls, with a ratio of either 1 to 3 or vice versa.

The first 16 trials were dedicated to a learning phase during which participants were informed that the computer would randomly select their choices. This was not the case, however, as the initial evidence was deliberately structured to induce a pseudocontingency inference. Specifically, one bag was randomly selected to be sampled 12 times, while the other bag was sampled 4 times. The ratio of blue and yellow balls presented in this stage was consistent with the true distribution for both bags (3:1); the bags were identical and the learning phase did not exhibit an actual contingency between the bags and the colors of the balls. Nevertheless, we expected that participants would form a pseudocontingency inference by associating the (in)frequent option with the (in)frequent outcome, resulting in the perception of a positive relationship between the most frequently displayed bag and the predominant color as well as an association between their infrequent counterparts, in line with previous research (cf. Fiedler et al., 2009).

After the learning phase, participants were prompted to provide estimates of the probabilities of drawing a ball of one of two colors from bag A, and from bag B. To indicate their estimations, they were provided with a slider that ranged from 0 to 100%. These premeasures served to assess whether the bias induction was successful.

##### Free-sampling phase

Prior to the free-sampling phase, an additional instruction was introduced, namely that from here on out one of the two colored balls (e.g., blue) would yield 10 points while the other colored balls (e.g., yellow) would have no consequence. In the FR condition, this meant that from here on out the more frequently encountered color would be rewarding. In the IR condition, this meant that from here on out the infrequently seen color would be rewarding. The instructions emphasized that the number of points gathered during this phase would determine the eventual payoff, stimulating participants to select their choices adaptively as to maximize the expected reward. During the remaining 84 trials, participants sampled freely from the two bags. The image of the winning ball was adjusted so that it displayed a ‘+10’. The current number of points earned was shown in the top right corner.

After these 84 trials, participants were asked to once more provide their probability estimates for drawing a specific color ball from each bag. These postmeasures served to assess the presence of a final bias. Finally, participants learned the total amount of money they had earned.

#### Data analysis

##### Data preparation

In line with the preregistration, participants who failed at least two of the three attention checks were excluded from the analysis (*n* = 61). It is important to note that this paper describes biases in choices as a predisposition towards the supposedly best option, based on the expected pseudocontingency inference from the initial learning phase. Hence, this is the most commonly displayed bag in the FR condition and the least frequently shown bag in the IR condition. Biases in estimates, however, are characterized in terms of the perceived relationship between the most frequent option and the most frequent outcome.

##### Conditional estimates

The conditional probability estimates of both the pre- and postmeasures were adjusted to account for counterbalancing, resulting in two variables, denoted ${P(Bal{l}_{f}|Bag}_{f})$ and ${P(Bal{l}_{f}|Bag}_{i})$. ${P(Bal{l}_{f}|Bag}_{i})$ represents the likelihood of drawing the most frequently observed ball given that the most frequently shown bag is chosen, while ${P(Bal{l}_{f}|Bag}_{i})$ represents the probability of drawing the same ball when the least frequent bag is chosen. We calculated the difference between these probabilities ∆P = ${P(Bal{l}_{f}|Bag}_{f})$ − ${P(Bal{l}_{f}|Bag}_{i})$ as a measure of contingency, representing the perceived dependence of obtaining the most prevalent outcome on choosing the bag that was presented most frequently (Allan, 1980). A two-sided *t*-test was conducted for each condition separately. Additionally, we conducted a *t*-test to compare the difference in contingency estimates between the two conditions.

##### Choice behavior

The dataset for the analysis of behavior included 84 observations for each of the 300 participants, each corresponding to one free-choice trial. We ran a mixed effects logistic regression with individual random effects to examine participants’ choice behavior. The outcome variable indicated whether participants had chosen the supposedly best option or the alternative (*1* = best; *0* = alternative), so that a significant effect would suggest a deviation from chance level in the probability of selecting the supposedly best option over the alternative. Trial number and condition (*1* = FR; *0* = IR) entered the model as explanatory variables, along with their interaction. To facilitate model convergence, the trial number was rescaled to a smaller range by subtracting 1 and dividing it by 83, so that the first trial was transformed to 0 and the last trial to 1. This rescaling set the beginning of the task as the baseline, with the intercept and condition coefficient reflecting the first trial. Additionally, we analyzed behavior at the end of the task as a baseline by reversing trial numbers so that the last trial was coded as 0 in the same mixed-effects model. To minimize Type-I and Type-II errors, we accounted for within-subject random effects by estimating the maximum feasible model, which included a random intercept and a random coefficient for trial number (Barr et al., 2013; Seedorff et al., 2019). Although a quadratic model was considered to allow for a non-linear effect of trial, the linear model was found to be superior based on the AIC. Models were estimated using the “lme4” R package (Bates et al., 2015) and diagnostics were assessed with the “performance” package (Lüdecke et al., 2021).

## Results

### Contingency estimates: premeasures

Conditional probability estimates were assessed to verify whether the predetermined learning phase resulted in a pseudocontingency inference. The estimated contingency between the most frequent bag and the predominant color ball was calculated using the *Δ * P measure. Despite the dispersion of individual estimates over positive and negative values, the average estimate for *Δ * P was significantly positive in both conditions. Participants in the IR condition reported an average contingency of *M* = .13, *SD* = .25, *t*(150) = 6.23, *p* < .001, while those in the FR condition reported *M* = .10, *SD* = .27, *t*(148) = 4.58, *p* < .001, on average. The difference between these averages was small and not statistically significant, ∆*P* = 0.11, *t*(298) = 0.92, *p* = .357.

### Choice behaviour

Participants’ choices were analyzed through a series of logistic mixed effect models. For the sake of parsimony, variance–covariance matrices are provided in Appendix B and only key findings are represented in-text to preserve legibility.

**Fig. 1:**
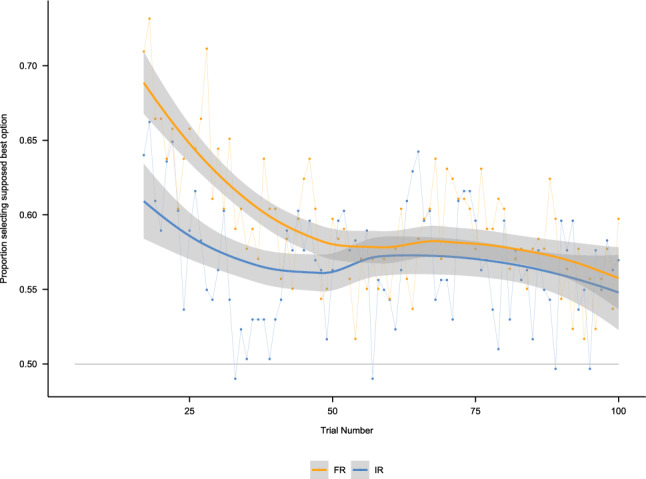
Proportion of choices for the supposed best option across trials in Experiment 1. *Note.* This figure represents the smoothed conditional means of the proportion of participants selecting the supposed best option in each trial. It was generated using the ‘geom_smooth()’ function from the R package ggplot2 (Whickham, 2016) and does not depict the linear models estimated for this dataset

Results of the baseline model are presented in Table 2. The intercept was positive and significant (*c* = 0.46, *p* = .003), which indicates that the probability to sample the supposed best option was initially above chance level for the IR condition in the first few trials.^3^ The positive simple effect for condition (*b* = 0.46, *p* = .035) suggests that this probability was higher for the FR group, in line with the second hypothesis. By the last trial, there was no longer a difference between conditions (*b* = −0.04, *p* = .931) and the baseline probability to sample the supposed best option had converged to chance level (*c* = 0.52, *p* = .083). Hence, despite the insignificance of the trial-by-trial difference, there was a substantial discrepancy between the first and the last trial. Correspondingly, Fig. 1 shows a large bias in choices across the initial trials, which was slightly higher in the FR condition. Choice proportions approached chance level after about one third of the trials, after which it fluctuated within a stable range in both groups. From this, it can be inferred that the nonsignificant coefficient for trial was likely due to non-linearity. Across trials, the overall proportion of choices for the supposed best option was significantly above chance level, with *M* = .59 (*d* = .33; *t*(148) = 4.02; *p* < .001) in the FR condition and *M* = .57 (*d* = .25; *t*(150) = 3.06; *p* = .003) for participants in the IR group.

**Table 2:** Regression models predicting choices in Experiment 1

	Full
Intercept	0.46
	*SE* = 0.153 *p* = .003
Condition	0.46
	*SE* = 0.22 *p* = .035
Trial	0.06
	*SE* = 0.26 *p* = .811
Condition × Trial	−0.50
	*SE* = 0.36 *p* = .171
Observations	25,198
Log Likelihood	−12,597.370
Akaike Inf. Crit	25,208.750
Bayesian Inf. Crit	25,265.690

Regressions included dummy variables for choice (*1* = supposed best option; *0* = alternative) and condition (*1* = frequent reinforcement; *0* = infrequent reinforcement)

We also conducted further exploratory analyses of the trial-by-trial choices. We found an overall preference to stay with a given option (as opposed to switching to the alternative). The IR condition initially switched more than the FR condition, though this tendency decreased over time. We report the analyses in Appendix C.

Taken together, these results are consistent with biased sampling in the first trials, which was more pronounced in the FR condition. Nevertheless, the bias and the difference between conditions had disappeared by the end of the free-sampling phase.

### Contingency estimates: postmeasures

**Fig. 2:**
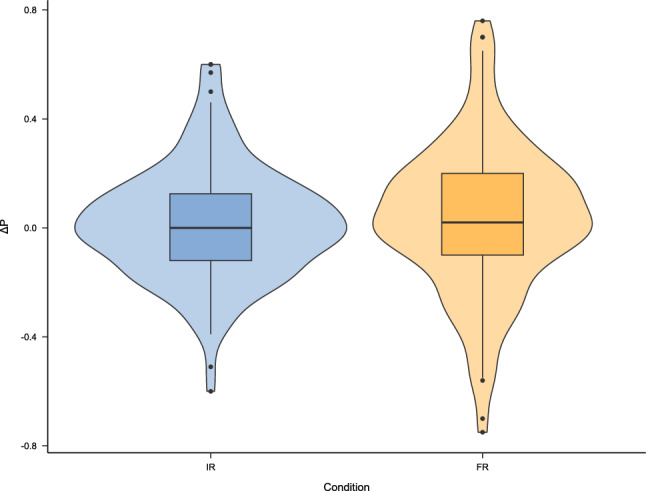
Distribution of final contingency estimates per condition in Experiment 1. *Note.* Contingency estimates were calculated for the strength of the association between the most frequent option and the most frequent outcome

After the free sampling phase, participants had revised their contingency estimates downwards. As expected, the IR group did no longer display a significant bias, with *M* = .02, *SD* = .21, *t*(150) = 0.99, *p* = .322. The estimate in the FR condition was slightly higher, though not significantly different from zero, *M* = .04, *SD* = .27, *t*(148) = 1.61, *p* = .109. Consequently, the difference between the two conditions was small and not significant, *d* = −0.08, *t*(276.04) = 0.69, *p* = .490, which is in contradiction with our third hypothesis. Figure 2 shows the distribution of the contingency estimates for each condition.

## Discussion

In conclusion, the results of this experiment provided only weak support for the proposed mechanism of bias persistence. As expected, participants made a pseudocontingency inference after the initial learning phase, supporting our first hypothesis and replicating prior evidence for pseudocontingency inferences as a source of illusory correlations. This bias affected initial choices, especially in the FR condition. However, whereas the second hypothesis suggested that uneven choice behavior would persist in the FR condition, behavioral biases attenuated over time in both conditions. Similarly, after the free sampling phase, the contingency estimates had turned nonsignificant in both conditions, thereby providing no support for the third hypothesis that beliefs would remain biased in the FR condition.

The absence of a persisting significant effect of condition on choices and beliefs is in contrast with the results of Harris et al. (2020). We made a couple of changes to this original paradigm. First, the total and completed numbers of trials were not indicated during the task. This introduces additional uncertainty regarding the available time for exploration and exploitation, which could affect the trade-off (Cohen et al., 2007). In addition, the present experiment contained a short game before the decision-making task, which may have contributed to boredom or fatigue. Although these small changes could in principle explain the difference in effects, we do not believe this very likely.

The biggest change, of course, was that the negative outcomes were replaced by neutral ones. The difference between the positive and neutral option may have been insufficient to induce reward-maximizing behavior, especially given the small range of additional (i.e. the incentivized part of the) earnings participants could receive for this task. While previous experiments were conducted with outcomes of −10 to +10, the current study may have resulted in a less pronounced contrast between choice alternatives. Moreover, there is substantial evidence that losses tend to be weighted more heavily than gains of the same magnitude (Kahneman & Tversky, 1979). Alternatively, previous results may have been primarily driven by avoidance of aversive consequences, rather than reward-maximization. Experiment 2 explores that possibility by implementing an aversive decision-making context.

## Experiment 2

### Methods

Experiment 2 aimed to test whether biased sampling and impressions persist when learning is driven by avoidance of aversive outcomes rather than the pursuit of rewards. Methods were pre-registered before the start of data collection (see https://aspredicted.org/7Z3_YKP) and were largely similar to those of Experiment 1. The only difference between the two studies was the incentivization. Whereas Experiment 1 implemented financial gains as a reinforcer, behavior in Experiment 2 was reinforced through omissions of an expected financial loss.

#### Participants

The participant recruitment procedure for Experiment 2 followed the same protocol as in Experiment 1. We deviated from the preregistration by including participants who did not exhibit the expected pseudocontingency bias, as we believed that considering the full distribution of initial biases would be more meaningful. The final sample consisted of *N* = 301 participants (${N}_{female}$= 153), with an average age of 32.05 years (*SD* = 10.79).

#### Design

The design was analogous to that of Experiment 1. However, the least desirable outcome in the current experiment entailed a negative consequence, resulting in a loss of 10 points, whereas the reinforcing outcome involved the avoidance of this loss (i.e., a neutral outcome).

#### Procedure

With some notable exceptions, the study proceeded in an identical manner to Experiment 1.

##### Induction of a loss frame

The purpose of this study was to investigate the trade-off between exploitation and exploration in the context of aversive learning by inducing a loss frame. For that purpose, participants were endowed with an initial amount of money that they would lose during the decision-making task.

Several measures were taken to increase the likelihood that participants would consider the initial endowment as a reference point and process losing points as an actual loss rather than the absence of a reward. Firstly, previous research has indicated that the explicit mention that an endowment is randomly determined tends to weaken attachment (Knetsch & Wong, 2009). Therefore, we aimed to create the impression that participants had earned the points because of their performance in a game. The initial task served that purpose as it was deliberately designed to hinder participants from accurately evaluating their own performance.

During the task, participants were presented with a screen displaying quickly flickering ‘X’s and ‘Y’s for a duration of 50s. Prior to this, they were informed that they would be required to estimate either the number of ‘X’s or the number of ‘Y’s that were displayed on the screen. As intended, the speed and frequency with which these letters were presented made it impossible to accurately track these numbers. The question (number of X’s or Y’s) was randomly determined for each participant, and they were asked to indicate their estimate on a scale of 0 to 100. Irrespective of the accuracy of their answer, participants were all awarded the same 843 points, which was explicitly stated to correspond to a financial reward of £5.

Physical possession was also found to play a significant role in determining the reference point (Knetsch & Wong, 2009). Since this is not possible in an online experiment, the study approximated this effect by including a clear display of the points earned throughout the remainder of the survey. Moreover, the time period between receiving the endowment and learning that it would be lost again was extended as much as possible (Song, 2016). We inserted the demographics questionnaire between the two tasks and individuals only learned that they would lose points after the learning phase of the second task. In the demographics questionnaire, participants were asked to indicate what they planned to do with the £5 they had earned. This question was added to increase the salience of the reward and the potential aversiveness of losing it (Shu & Peck, 2011).

##### Decision-making task

The bias induction proceeded identically to Experiment 1. Afterwards, participants in the IR condition were informed that the most frequent ball would result in a loss of 10 points, whereas the less frequent ball would remain neutral. The opposite held for participants in the FR condition. The instructions emphasized that their current reward of £5 would shrink as a function of the number of points they would lose during the task.

During the subsequent 84 trials, participants freely sampled from the two bags. The trial structure remained the same as the learning trials, except for the image of the losing ball, which displayed a ‘−10’ in order to strengthen the loss frame. In the top right corner of the page, the points obtained from the preceding game as well as the points that were lost during the task were displayed. The remaining points were displayed prominently in bold font, centered at the top of the page.

After the final measurement of the conditional probability estimates at the end of the task, participants completed a questionnaire that contained a series of scaled-response questions, evaluated on a 7-point Likert scale (*0* = strongly disagree; *6* = strongly agree). The first two items assessed participants’ attachment to the £5 they had supposedly earned in the beginning and were adopted from the psychological ownership questionnaire (Shu & Peck, 2011). The purpose of this was to confirm that the reference point manipulation was effective. In addition, five questions gauged the aversiveness of losing points in the second task, and three statements measured participants’ subjective valuation of a £5 reward. These sets of questions were devised ad hoc and included as a precautionary measure to check for any potential inconsistencies that could be attributed to not experiencing aversion or to a differential subjective valuation of the same monetary amount. Afterwards, the total earnings were revealed, which again ranged between £1.5 and £2.5.

#### Data analysis

The data analysis for this study followed the same protocol as Experiment 1. In addition, summary statistics were computed for the results of the final questionnaire and two-sided *t-*tests were performed to determine whether Likert responses deviated significantly from the neutral response.

## Results

### Contingency estimates: premeasures

The premeasures for conditional probability estimates showed a successful bias induction. The participants’ average estimated contingencies after the learning phase were positive and significant, equaling *M* = .138, *SD* = .27, *t*(153) = 6.30, *p* < .001, in the IR condition and *M* = .086, *SD* = .28, *t*(146) = 3.68, *p* < .001, in the FR condition, in line with our hypothesis. The difference in estimates between the two conditions was not statistically significant, ∆*P* = 0.19, *t*(299) = 1.61, *p* = .108.

### Choice behavior

**Fig. 3:**
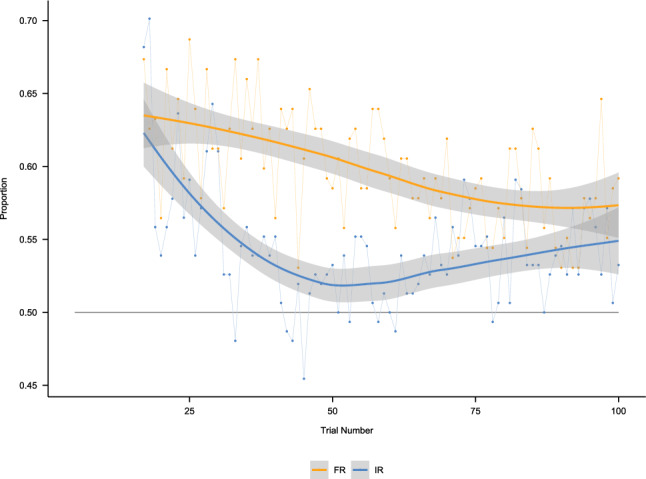
Proportion of choices for the supposed best option across trials in Experiment 2. *Note.* This figure represents the smoothed conditional means of the proportion of participants selecting the supposed best option in each trial. It was generated using the ‘geom_smooth()’ function from the R package ggplot2 (Whickham, 2016) and does not depict the linear models estimated for this dataset

Choices during the free-sampling phase showed a clear pattern, depicted in Fig. 3: Whereas initial sampling behavior was biased in both conditions, the IR condition adjusted their choices much more quickly than participants in the FR condition. This was supported by the regression results reported in Table 3. Similar to Experiment 1, we refer to the tables for more detailed results than those included in-text. Initially, the probability to choose the supposed best option was significantly above chance level for participants in the IR condition (*c* = 0.34, *p* = .022) and was even higher in the FR condition (*b* = 0.56, *p* = .009). There was no significant main effect of trial (*b* = −0.13, *p* = .548) nor any interaction with condition (*b* = −0.06, *p* = .848). However, by the final trial, the overall probability of choosing the supposed best option had converged to chance level, indicated by the non-significant intercept (*c* = 0.20, *p* = .458) and the condition coefficient (*b* = 0.50, *p* = .208). Similar to Experiment 1, the overall proportion of choices in favor of the supposed best option was significantly larger than chance level in both the FR (*M* = .60; *d* = .33; *t*(146) = 4.03; *p* < .001) and the IR group (*M* = .54; *d* = .16; *t*(153) = 2.04; *p* = .04).

**Table 3:** Regression models predicting choices in Experiment 2

	Full
Intercept	0.34
	*SE* = 0.15 *p* = .022
Condition	0.56
	*SE* = 0.21 *p* = .009
Trial	−0.13
	*SE* = 0.22 *p* = .548
Condition × Trial	−0.06
	*SE* = 0.33 *p* = .848
Observations	25,284
Log Likelihood	−12,817.250
Akaike Inf. Crit	25,648.490
Bayesian Inf. Crit	25,705.460

Regressions included dummy variables for choice (*1* = supposed best option; *0* = alternative) and condition (*1* = frequent reinforcement; *0* = infrequent reinforcement)

The exploratory analyses indicated an increasing tendency to explore in the IR condition as the trials progressed. Conversely, in the FR condition, the likelihood of sticking with a single option increased over time. For additional details, please refer to Appendix C.

### Contingency estimates: postmeasures

**Fig. 4:**
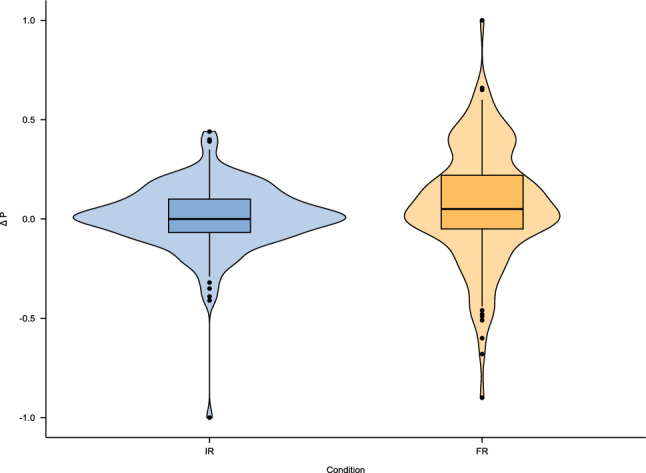
Distribution of final contingency estimates per condition in Experiment 2. *Note.* Contingency estimates were calculated for the strength of the association between the most frequent option and the most frequent outcome

Following the free sampling phase, participants in the IR condition adjusted their contingency estimates towards a value of 0, with an estimated mean of *M* = .01, *SD* = .17, *t*(153) = 0.61, *p* = .541. In contrast, individuals who encountered infrequent aversive outcomes (FR condition) still showed a significant bias, with *M* = .07, *SD* = .29, *t*(146) = 2.98, *p* = .003. Moreover, the difference between conditions was significant, ∆*P* = −0.27, *t*(233.05) = 2.28, *p* = .023. Distributions of the contingency estimates are depicted in Fig. 4.

### Discussion

Contrary to Experiment 1, conditional probability estimates in Experiment 2 provided support for both the first and third hypothesis. That is, the uneven presentation of evidence in the learning phase successfully induced an initial association between the most frequent option and most prevalent outcome, and this relationship persisted after free sampling in the FR condition. As such, the results indicated that the frequency of reinforcement significantly affected bias persistence. While both groups adjusted their contingency estimates, the average in the FR condition was still significantly larger than zero. Results for choice behavior were largely consistent with our expectations. Initial choices were biased, especially in the FR condition. In the last trials there was no significant difference in the probability to sample the supposed best option between conditions. Nevertheless, participants in the FR condition were still more likely to select the supposed best option as opposed to the alternative, whereas no such tendency was detected in the IR condition.

Overall, the second experiment provided evidence that the mechanism demonstrated by Harris et al. (2020) generalizes to an aversive environment, despite some ambiguity in choice behavior.

## General discussion

The objective of this study was to examine how positive and negative outcomes contribute to the persistence of biased impressions and choice behavior in rewarding and aversive contexts, respectively, using a two-armed bandit task. The first hypothesis posited that the uneven presentation of initial evidence would cause the frequent option to be associated with the predominant outcome while the infrequent alternative would be linked to the infrequent outcome, despite the actual action-outcome contingency being zero. Second, it was expected that the influence of these pseudocontingency inferences on sampling behavior would persist in the frequent reinforcement condition and attenuate for participants receiving infrequent reinforcement. Third, we suggested that continued biased sampling in the frequent reinforcement condition would translate into biases in explicit contingency estimates. That is, we anticipated that participants in the IR condition would have adapted their estimates to represent the absence of any contingency, while those in the FR condition would not.

### Summary of the results

Taken together, this research provides some evidence in support of the proposed mechanism of bias perpetuation. The bias induction (first hypothesis) was successful in both experiments, while the perpetuation of illusory correlations (second and third) was observed only in Experiment 2 (negative vs. neutral outcomes). Here, we found that the frequent omission of financial losses contributed to the persistence of negative impressions of the least frequently presented option, both in behavior and in later estimates.

### Interpretation

Overall, there are two main take-aways from our experiments. First, our results suggest that the paradigm proposed by Harris et al. (2020) also applies to environments that lack rewards. Frequent reinforcements resulted in persisting biases when outcomes in the environment were exclusively negative or neutral. Second, our results, and especially the results from Experiment 1 (positive vs. neutral outcomes), suggest that carefully differentiating between positive, aversive, and neutral outcomes is warranted, even when participants’ goal remains reward maximization.

It is intriguing that final biases seemed to persist in the aversive experiment (Experiment 2) but not in the rewarding experiment (Experiment 1). As these studies were nearly identical, the absence of an effect in the rewarding variant is most plausibly related to the elimination of negative outcomes. Potentially, this absence of losses resulted in an insufficient incentivization to encourage reward-seeking behavior compared to previous experiments (cf. Harris et al., 2020). More specifically, either losing or winning might have led to a much starker contrast between the potential outcomes, and hence a stronger incentive to exploit the supposedly best option. In our Experiment 1, positive outcomes or a neutral alternative might have produced a weaker contrast and hence a weaker incentive to exploit. Interestingly then, loss aversion might be part of the reason why we still found persisting biases in Experiment 2: While the contrast again was much weaker (negative vs. neutral as opposed to negative vs. positive), this contrast might have still been perceived as stronger than the contrast between positive and neutral outcomes. It could be that we chanced on the tipping point where the contrast in outcomes was sufficient to encourage exploitation (Experiment 2) or not (Experiment 1).

Another, complementary interpretation is that biases in previous experiments were driven by negative outcomes. The hypothesized effect hinges critically on the assumption that participants attribute the observed outcome to their choices rather than to the general distribution of outcomes across the decision-making context. Therefore, the results may suggest that this attribution of feedback to specific responses was more likely to occur in aversive learning contexts, while participants in the rewarding experiment might have interpreted the outcomes as being representative of the task in general.

While both FR conditions were equal in terms of reinforcement rates, they were opposites with regard to the representation of the focal stimuli. That is, the experiments not only differed in the valence of the incentives used, they also differed as to whether the preferred outcome involved the *presence* or *absence* of that incentive, potentially affecting the way in which the sampled information was processed (Elliot et al., 2024). Similarly, learning research postulates that extinction learning is more likely to be attributed to situation-specific stimuli than an unexpectedly high frequency of an expected effect (Lucke et al., 2013; Vervliet et al., 2013). As such, frequent positive reinforcement in Experiment 1 may have strengthened overall reward expectations, whereas in Experiment 2, frequent omission of an expected aversive event may have functioned similarly to threat extinction. In this context, bias persistence in the aversive experiment might reflect a protection from extinction effect. This means that individuals whose threat expectancy was higher than the base rate of negative outcomes would have failed to update their general expectations as they attributed the omission of threat to their choice for the supposed best option, resulting in persisting negative impressions of the alternative (Lovibond et al., 2009). In the IR condition, the frequent administration of an aversive outcome (Experiment 2) would merely have strengthened participants’ expectations that the task was aversive. Similarly, in a rewarding context (Experiment 1), participants in the FR condition may have faced reinforcement of exploitation but the differential effect on belief-updating can account for the limited effect on beliefs. Moreover, participants in the IR condition would be subject to extinction if rewards occur less frequently than they expected. In contrast with the aversive experiment, however, attribution of the absence of rewards to their response would then cause them to sample the alternatives, which allows for updating of expectations of the environment in general. The finding that biases in beliefs were much more pronounced than biases in choice behavior is consistent with a differential effect on belief-updating as a plausible explanation.

While our results highlight the importance of carefully differentiating between the effects of positive and of negative outcomes, they do not rule out that inaccurate beliefs can also persist through the pursuit of pleasurable experiences. The elimination of negative outcomes in our Experiment 1 introduced differences compared to previous research, beyond merely isolating the hedonic motive underlying choices. First, the contrast between obtaining a reward and receiving no reward was more subtle, both in terms of the quantitative difference in points participants could earn and the qualitative difference between outcomes. It is plausible that the contrast between winning and not winning triggers different framing effects than the contrast between winning and losing. This increased subtlety may have reduced the influence of the hedonic motive, leading to a lack of observable effects on information sampling, especially given the simplicity of the task; as the potential for biases tends to increase with the complexity of the decision-making context. Additionally, the focus on ‘winning’ or ‘not losing’, and the accompanying expectations may have elicited different regulatory foci (cf. Higgins, 1998) or information processing modes (cf. Bless & Fiedler, 2006; Forgas & Eich, 2013), affecting judgments and decisions. These considerations suggest that seemingly similar settings may activate different mechanisms. In summary, this study represents a preliminary attempt to disentangle the effects of approaching a desirable option from avoiding an undesirable one. Future research could further explore these underlying mechanisms. The fact that a seemingly minor adjustment had significant consequences, with numerous potential explanations, underscores the importance of distinguishing between sampling for rewards and sampling to avoid aversive events.

### Implications

The present research explored how reinforcement learning, typically viewed as adaptive, may become maladaptive due to an interaction with the distribution of outcomes and information in the decision-making environment (Fiedler, 2000; Rich & Gureckis, 2018). Similar to Harris et al. (2020), our findings indicate that the combination of a choice-dependent information sample and a hedonic motive is sufficient to override the effect of genuine contingencies on behavior and judgment. However, in contrast to previous studies that focused on the pursuit of rewards contrasted against losses, this research emphasizes the significance of differentiating between sampling to pursue positive outcomes and sampling to prevent negative outcomes. Our findings suggest that aversive environments may be particularly prone to bias persistence. This has implications across theoretical and practical domains, highlighting the importance of attribution errors and their impact on action selection and learning.

#### Discrimination

This research extends the literature on illusory correlations, which are particularly relevant to stereotyping and discrimination, by integrating principles of research on learning. Interventions that focus on minimizing the occurrence of minority group discrimination could benefit from this framework in several ways. The biased sampling account speaks for the effectiveness of minority group quotas and affirmative action in order to increase their representation in society (Denrell, 2005; Fazio et al., 2004; Le Mens & Denrell, 2011). While we do not contest the importance of such measures, aversive discriminatory attitudes would not be eliminated if positive information is attributed to specific co-occurrences. For instance, prejudiced individuals who get acquainted with a friendly minority group member would attribute their friendliness to their personality, rather than their group membership (cf. Pettigrew, 1979; Weber & Crocker, 1983) Hence, while quotas are essential to minimize the unevenness of samples, it would not be sufficient to counter all types of biases. The current study suggests that providing information about negative attributes from majority group members that are typically associated with minorities may, in some cases, be more productive than providing individuals with positive information about minority groups. This fosters the realization that a behavioral bias against minorities does not minimize the probability of having an aversive experience. Lastly, the unstable nature of extinction means that negative group-attribute associations would be difficult to overcome. Therefore, it may be more productive to focus on categorization itself, rather than addressing the impressions people hold of a specific group. This could be done through interventions that focus on increasing the salience of similarities, rather than emphasizing the differences, in an attempt to have minorities be perceived as a part of the majority through focusing on inclusiveness (Brewer, 2016).

#### Anxiety

The finding that the adaptiveness of experiential learning is obscured by attempts to minimize the probability of an aversive event is particularly relevant to research on irrational fears and clinical anxiety. Given the similarities between hedonic sampling in an aversive context and *safety behaviors* (cf. De Houwer & Hughes, 2020; Krypotos et al., 2015; Rattel et al., 2017), the current approach can address some of the limitations of existing fear conditioning paradigms by incorporating ambiguity and active engagement with the environment into the learning context. This allows for an impact of individual decision-making processes on fear acquisition, which could account for the empirical findings that individuals with similar experiences exhibit diverse outcomes in terms of anxiety (Beckers et al., 2013). This is illustrated by the variation in individual contingency estimates within conditions. In summary, accounting for the bidirectional relationship between learning and behavior can enhance the ecological validity of the fear conditioning paradigm and, as such, provide important insight into how irrational fears and maladaptive avoidance develop and persist in the real world (Pittig et al., 2018).

If replicable with fear-related stimuli, our approach could have various implications for the treatment of clinical anxiety. The difference between conditions implies that the extinction of safety behaviors could be a promising way to combat clinical anxiety, especially when complemented with Pavlovian fear extinction through exposure therapy. Since this requires the execution of safety behaviors being followed by the feared aversive event, this may not be attainable for all types of fears. Nevertheless, research on derived relational responding suggests that similar effects could be obtained with talk-based therapies (Dymond & Roche, 2009). This would moreover eliminate the paradox that arises because the unavailability of safety responses is a contextual factor under extinction learning, while the presence of such responses inhibits extinction learning (Treanor & Barry, 2017). Additionally, the importance of hedonic motivations implies that reframing the goal from being comfortable to learning to live with the occasional presence of anxiety and aversiveness could prove beneficial in order to tackle generalized avoidance.

### Limitations

Like any research endeavor, this study is not without its limitations. Therefore, it is advisable to interpret the findings with due caution.

Several factors may compromise the generalizability of our results. The use of monetary incentives calls for further investigation to determine whether the observed effects translate to other rewarding and aversive outcomes. Moreover, the choice paradigm used in this study, consisting of 84 sequential binary decisions, may not accurately mirror real life decision-making scenarios, which tend to involve more options and greater spatiotemporal dispersion. However, we expect biases to be more pronounced under more realistic conditions or with the use of fear-inducing aversive stimuli. Lastly, our inclusion criteria likely resulted in the recruitment of a specific subsample of the population, so that the generalizability of our results across individuals is limited.

Furthermore, the experiments conducted do not offer a direct comparison between positive and negative decision-making contexts, since the experiments were conducted independently. While the diversity of online samples make it unlikely that specific circumstances affected the experiments systematically, nonetheless caution should be exercised when comparing them directly.

Finally, it is important to recognize that the study assumed participants were primarily motivated by hedonic factors. This assumption aligns with the emphasis on financial incentives throughout the experiment and the fact that participants were recruited from a platform where they specifically signed up to earn money by taking part in studies. While we did not explicitly assess their motivations and cannot completely dismiss alternative motives, such as positive testing strategies (Denrell & Le Mens, 2011; Harris et al., 2020; Klayman & Ha, 1987), the hedonic motive appears to be the most plausible and parsimonious explanation.

## Conclusion

In conclusion, this research sought to explore the ways in which reinforcement learning can propagate biases that lead to maladaptive behaviors. While hedonic sampling theories predict similar outcomes for both positive and negative situations, we highlighted the importance of distinguishing between rewards and aversive outcomes. Our findings suggest that the effect of hedonic sampling is particularly pronounced in aversive environments, where frequent reinforcement results in the persistence of initial biases. We suggested that this is due to the context-dependence of extinction and the resulting misattribution of a desired outcome to a specific response in the aversive context. These results have important implications for social psychological research and learning, particularly in the context of maladaptive avoidance.

The current research primarily focused on the negative consequences of sampling biases and information asymmetries, but there are also benefits to these mechanisms in many situations. In environments with numerous possibilities, having continued unfavorable impressions of unexplored options can be advantageous to prevent inertia caused by anticipated regret. Moreover, judgments and decisions based on limited information can enhance individuals’ capacity to actively and flexibly engage with their environment. As such, the ability to navigate the complexity of our intersubjective reality and the detrimental consequences of biased beliefs may just be two sides of the same coin.

## Data Availability

The data and analysis scripts supporting the findings of this study are openly available on the Open Science Framework: https://osf.io/qrvz2/?view_only=ab10c5fd4c224b8d959089d855eb650c

## Appendix A: List of countries

To avoid excessive discrepancies in the valuation of monetary incentives, our inclusion criteria included residency in one of the following countries, listed in alphabetical order: Australia, Austria, Belgium, Canada, Denmark, Estonia, Finland, France, Germany, Iceland, Ireland, Italy, Luxembourg, the Netherlands, New Zealand, Norway, Spain, Sweden, Switzerland, United Kingdom, United States.

## Appendix B: Random effects standard deviations and correlations

See Tables

**Table 4:** Variance–covariance matrix for baseline model Experiment 1 (Table 2)

	Intercept	Trial	Condition	Condition × Trial
Intercept	0.023	0.001	−0.023	−0.001
Trial	0.001	0.065	−0.001	−0.065
Condition	−0.023	−0.001	0.048	0.001
Condition × Trial	−0.001	−0.065	0.001	0.133

^4^,

**Table 5:** Variance–covariance matrix for exploratory Model A Experiment 1(Table C1, Column 1)

	Intercept	Choice at *t–1*
Intercept	0.012	−0.017
Choice at *t–1*	−0.017	0.033

^5^,

**Table 6:** Variance–covariance matrix for exploratory Model B Experiment 1 (Table C1, Column 2)

	Intercept	Choice	Trial	Condition	Choice × Trial	Choice × Condition	Trial × Condition	Choice × Trial × Condition
Intercept	0.025	−0.034	−0.009	−0.023	0.008	0.032	0.007	−0.008
Choice	−0.034	0.066	0.009	0.032	−0.014	−0.063	−0.009	0.014
Trial	−0.009	0.009	0.023	0.007	−0.019	−0.009	−0.020	0.017
Condition	−0.023	0.032	0.007	0.052	−0.008	−0.069	−0.017	0.018
Choice × Trial	0.008	−0.014	−0.019	−0.008	0.034	0.014	0.017	−0.031
Choice × Condition	0.032	−0.063	−0.009	−0.069	0.014	0.133	0.020	−0.031
Trial × Condition	0.007	−0.009	−0.020	−0.017	0.017	0.020	0.043	−0.036
Choice × Trial × Condition	−0.008	0.014	0.017	0.018	−0.031	−0.031	−0.036	0.066

^6^,

**Table 7:** Variance–covariance matrix for exploratory Model C Experiment 1 (Table C1, Column 3)

	Intercept	Contingency		Choice		Trial		Condition		Choice × Trial			Choice × Condition		Trial × Condition		Choice × Trial × Condition
Intercept	0.026	−0.001		−0.035		−0.009		−0.024		0.008			0.032		0.007		−0.008
Contingency	−0.001	0.195		0.015		0.001		0.000		0.001			−0.006		−0.001		0.002
Choice	−0.035	0.015		0.067		0.009		0.032		−0.015			−0.063		−0.009		0.014
Trial	−0.009	0.001		0.009		0.024		0.007		−0.020			−0.009		−0.20		0.017
Condition	−0.024	0.000		0.032		0.007		0.052		−0.008			−0.070		−0.017		0.018
Choice × Trial	0.008	0.001		−0.015		−0.020		0.008		0.035			0.015		0.018		−0.032
Choice × Condition	0.032	−0.006		−0.063		−0.009		0.070		0.015			0.133		0.020		−0.032
Trial × Condition	0.007	−0.001		−0.009		−0.020		−0.017		0.018			0.020		0.043		−0.037
Choice × Trial × Condition	−0.008	0.002		0.014		0.017		0.018		−0.032			−0.032		−0.037		0.067

^7^,

**Table 8:** Variance–covariance matrix for baseline model Experiment 2 (Table 3)

	Intercept	Trial	Condition	Condition × Trial
Intercept	0.022	0.001	−0.021	−0.002
Trial	0.001	0.050	−0.002	−0.050
Condition	−0.021	−0.002	0.045	0.002
Condition × Trial	−0.002	−0.050	0.002	0.107

^8^,

**Table 9:** Variance–covariance matrix for exploratory Model A Experiment 2 (Table C2, Column 1)

	Intercept	Choice at *t–1*
Intercept	0.011	−0.013
Choice at *t–1*	−0.013	0.027

^9^,

**Table 10:** Variance–covariance matrix for exploratory Model B in Experiment 2 (Table C2, Column 2)

	Intercept	Choice	Trial	Condition	Choice × Trial	Choice × Condition	Trial × Condition	Choice × Trial × Condition
Intercept	0.019	−0.023	−0.006	−0.017	0.007	0.020	0.005	−0.007
Choice	−0.023	0.047	0.008	0.020	−0.013	−0.044	−0.007	0.013
Trial	−0.006	0.008	0.021	0.005	−0.016	−0.008	−0.019	0.014
Condition	−0.017	0.020	0.005	0.041	−0.007	−0.049	−0.012	0.016
Choice × Trial	0.007	−0.013	−0.016	−0.007	0.029	0.013	0.014	−0.026
Choice × Condition	0.020	−0.044	−0.008	−0.049	0.013	0.100	0.016	−0.028
Trial × Condition	0.005	−0.007	−0.019	−0.012	0.014	0.016	0.046	−0.034
Choice × Trial × Condition	−0.007	0.013	0.014	−0.016	−0.026	−0.028	−0.034	0.060

^10^, and

**Table 11:** Variance–covariance matrix for exploratory Model C in Experiment 2 (Table C2, column 3)

	Intercept	Contingency	Trial		Choice		Condition		Contingency × Trial		Trial × Choice		Choice × Condition		Trial × Condition		Trial × Choice × Condition	
Intercept	0.019	−0.004	−0.006		−0.023		−0.017		−0.002		0.007		0.021		0.005		−0.007	
Contingency	−0.004	0.224	−0.005		0.008		−0.004		−0.286		0.003		0.003		0.002		0.001	
Trial	−0.006	−0.005	0.021		0.009		0.006		0.002		−0.016		−0.009		−0.018		0.014	
Choice	−0.023	0.008	0.009		0.047		0.020		0.001		−0.013		−0.043		−0.008		0.013	
Condition	−0.017	−0.004	0.006		0.020		0.041		0.002		−0.007		−0.049		−0.013		0.016	
Contingency × Trial	−0.002	−0.286	0.002		0.001		0.002		0.915		−0.004		0.000		−0.001		−0.002	
Trial × Choice	0.007	0.003	−0.016		−0.013		−0.007		−0.004		0.029		0.013		0.013		−0.026	
Choice × Condition	0.021	0.003	−0.009		−0.043		−0.049		0.000		0.013		0.099		0.019		−0.028	
Trial × Condition	0.005	0.002	−0.018		−0.008		−0.013		−0.001		0.013		0.019		0.044		−0.033	
Trial × Choice × Condition	−0.007	0.001	0.014		0.013		0.016		−0.002		−0.026		−0.028		−0.033		0.060	

^11^.

## Appendix C: Exploratory analysis

### Experiment 1

#### Methods

To acquire a deeper understanding of the results and the underlying mechanisms, we performed additional exploratory analyses. First, choices in a given trial were regressed on the choice and outcome (*1* = win; *0* = neutral) from the previous trial, including random effects in the intercept and the lagged choice coefficient. Random effects for the lagged outcome were dropped due to singularity issues. Since there were no a priori expectations, all possible combinations of these explanatory variables were tested and compared using the AIC, BIC and likelihood-ratio tests. Subsequently, we added trial, condition and their interaction to the resulting model in all possible combinations and again selected the model with the best fit. In addition, we constructed a continuous variable that indicated the actual contingency that participants had encountered, based on the choices and the observed outcome for each preceding trial. This variable was included in the model to control for the extent to which behavior was influenced by actual contingency inferences.

#### Results

In our exploratory analyses, we aimed to gain more insight into the choice dynamics and evaluated whether the absence of an effect could be explained by a failure to induce increased exploitation of the supposed best option in the FR condition. Simultaneously, we also tested whether participants used a win-stay lose shift-strategy by examining whether the obtained outcome had an effect on the subsequent choice. That is, we estimated a series of binomial mixed effects regressions that regressed the choice in a given trial on the choice and outcome of the previous trial. Random effects were included on the intercept and on the coefficient for previous choice, while those pertaining to the lagged outcome were dropped because of singularity issues. This implies that we did not account for individual variability concerning reactions to outcomes. The analysis proceeded by estimating models with all possible fixed effects combinations and selecting those with the best fit according to the AIC and BIC, or a likelihood-ratio test when the first two criteria were inconclusive.

The outcome of the previous trial was consistently insignificant as a predictor. Therefore, the information criteria favored the model that merely included previous choice as an independent variable. This model is displayed in the first column of Table 3. The combination of a positive significant coefficient (*b* = 2.92, *p* < .001) and a negative significant intercept (*c* = −1.17, *p* < .001) suggests a substantial tendency to repeat the same choice. Moreover, the magnitude of the coefficient compared to the intercept indicates that participants were less likely to switch after they had chosen the supposed best option. Adding trial and condition as explanatory variables did not substantially affect these results, as indicated by Model B in Table 3. Remarkably, the effect of condition was no longer significant (*b* = −0.16, *p* = .484) due to its significant interaction with choice (*b* = 0.72, *p* = .047), revealing that the probability of switching was lower for the FR group in the first trials, in line with the proposed mechanism. Nevertheless, the interaction between the choice variable and trial number (*b* = 0.54, *p* = .003) signals that the tendency to switch in the IR condition decreased over time. As demonstrated in Model C, these findings were robust to adding the evidence encountered up until now, represented by a contingency measure calculated from participants’ previous choices and observed outcomes, which had a positive and significant coefficient (*b* = 2.30, *p* < .001).

#### Discussion

A closer inquiry into the decision-making dynamics revealed that choices were tremendously monotone. The probability to switch was generally low and was not affected by the outcome of the previous choice. This can reasonably be explained by taking into account that uncertainty was expected. Interestingly, individuals were more likely to repeat their previous choice if they had chosen the supposed best option, especially in the FR condition. As the main effect of condition disappeared after previous choice was accounted for, we can infer that the initial difference between conditions in the original model was driven by a higher probability to repeat the previous choice in the FR condition, in line with premature exploitation due to the reinforcement rate. Moreover, these results extended beyond the effect of actual contingencies on choices. Thus, while actual contingency does affect behavior to some degree, as can be expected from reinforcement learning, there is some evidence that its impact is obscured by initial impressions and sampling effects.

### Experiment 2

#### Results

We again tested whether participants used a win-stay lose shift-strategy by examining whether the obtained outcome had an effect on the subsequent choice. The results for the choice dynamics were largely similar to the Experiment 1, with some exceptions. It is clear from Model A in Table 6 that outcome did not contribute to explaining choices and that the preferred model again consisted merely of an intercept and the choice in the previous trial. Individuals tended to repeat their previous choices (i.e., stay) relatively frequently, as suggested by the negative intercept (*c* = −0.84, *p* < .001) and the positive coefficient for choice (*b* = 2.21, *p* < .001). These effects remain when trial number and condition are added to the model, displayed as Model B in the Table 6. Over time, there was a slight increase in the tendency to switch away from the supposed worst option in the IR condition, though the coefficient was insignificant (*b* = 0.28, *p* = .057). In combination with the negative interaction of previous choice and trial (*b* = −0.59, *p* < .001), pointing towards a similar increase in switches away from the supposed best option, this suggests that exploration increased in the IR condition as time went on. The reverse was found for the FR condition, where the probability to stick with one option increased over time, irrespective of whether this was the supposed best (*b* = 0.79, *p* = .001) or the supposed worst option (*b* = −0.53, *p* = .013). Model C showed that the experienced contingency was a significant predictor of choices as well (*b* = 2.93, *p* < .001), though its impact decreased over trials (*b* = −2.33, *p* = .015) and did not seriously affect other results (Tables

**Table 12:** Exploratory regression models predicting choices at time t in Experiment 1

	Model A	Model B	Model C
Intercept	−1.17 *SE* = 0.11 *p* < .001	−0.89 *SE* = 0.16 *p* < .001	−0.85 *SE* = 0.16 *p* < .001
Contingency			2.30
			*SE* = 0.44 *p* < .001
Choice at *t–1*	2.92	2.32	2.32
	*SE* = 0.18 *p* < .001	*SE* = 0.26 *p* < .001	*SE* = 0.26 *p* < .001
Trial		−0.25	−0.27
		*SE* = 0.15 *p* = .096	*SE* = 0.15 *p* = .081
Condition		−0.16	−0.19
		*SE* = 0.23 *p* = .484	*SE* = 0.23 *p* = .410
Choice at *t–1* × Trial		0.54	0.57
		*SE* = 0.18 *p* = .003	*SE* = 0.19 *p* = .002
Choice at *t–1* × Condition		0.72	0.74
		*SE* = 0.37 *p* = .047	*SE* = 0.36 *p* = .042
Trial × Condition		−0.18	−0.17
		*SE* = 0.21 *p* = .394	*SE* = 0.21 *p* = .406
Choice at *t–1* × Trial × Condition		−0.32	−0.36
		*SE* = 0.26 *p* = .220	*SE* = 0.26 *p* = .168
Observations	24,898	24,898	24,734
Log Likelihood	−10,434.84	−10,409.13	−10,295.19
Akaike Inf. Crit	20,879.68	20,846.26	20,628.39
Bayesian Inf. Crit	20,920.29	20,959.97	20,782.59

Regressions included dummy variables for choice (*1* = supposed best option; *0* = alternative) and condition (*1* = frequent reinforcement; *0* = infrequent reinforcement)

12,

**Table 13:** Exploratory regression models predicting choices in Experiment 2

	Model A	Model B	Model C
Intercept	−0.84	−0.92	−0.89
	*SE* = 0.11 *p* < .001	*SE* = 0.14 *p* < .001	*SE* = 0.14 *p* < .001
Contingency			2.93
			*SE* = 0.47 *p* < .001
Choice at *t–1*	2.21	2.15	2.14
	*SE* = 0.16 *p* < .001	*SE* = 0.22 *p* < .001	*SE* = 0.22 *p* < .001
Trial		0.28	0.23
		*SE* = 0.15 *p* = .057	*SE* = 0.14 *p* = .107
Condition		0.31	0.24
		*SE* = 0.20 *p* = *.*132	*SE* = 0.20 *p* = .242
Choice at *t–1* × Trial		−0.59	−0.55
		*SE* = 0.17 *p* < .001	*SE* = 0.17 *p* = .001
Contingency × Trial			−2.33
			*SE* = 0.96 *p* = .015
Choice at *t–1* × Condition		0.08	0.12
		*SE* = 0.32 *p* = .797	*SE* = 0.31 *p* = .712
Trial × Condition		−0.53	−0.45
		*SE* = 0.21 *p* = *.*013	*SE* = 0.21 *p* = .034
Choice at *t–1* × Trial × Condition		0.79	0.76
		*SE* = 0.25 *p* = .001	*SE* = 0.25 *p* = .002
Observations	24,983	24,983	24,983
Log Likelihood	−11,415.60	−11,387.07	−11,361.32
Akaike Inf. Crit	22,841.19	22,802.14	22,762.65
Bayesian Inf. Crit	22,881.82	22,915.91	22,925.17

Regressions included dummy variables for choice (*1* = supposed best option; *0* = alternative) and condition (*1* = frequent reinforcement; *0* = infrequent reinforcement). Contingency was calculated for the relationship between the supposed best option and the desired outcome

13).

#### Discussion

Similar to Experiment 1, switches were relatively rare. While there were no initial differences between conditions regarding switches, the tendency to stick with one choice increased for participants who received frequent reinforcement and decreased for the other group. This is consistent with the reinforcement of exploitation in the FR condition and absence thereof in the IR condition. Finally, increased exploitation in the FR environment, combined with a decreased probability to sample the supposed best option, indicates that some individuals developed sampling biases in favor of the supposed worst option, which was also reflected in the contingency estimates.

## General discussion

Participants rarely switched between alternatives and their choices were unrelated to the outcome of the previous trial. Those who had chosen the supposed best option in the preceding trial were even more likely to repeat their choice, especially in the FR conditions. Additionally, in the aversive experiment, the probability of switching decreased over time in the FR condition and increased in the IR condition, indicating a divergence between conditions. The opposite held true for the rewarding experiment, where the probability of switching showed an increasing trend in the FR condition and declined over time in the IR group.

## Footnotes

1 Note that even such a strategy can result in biased samples (Fiedler, 1996).

2 It is important to note that the sequence in which the experiments are presented in this paper does not match the actual sequence in which they were conducted. Experiment 2 was performed before Experiment 1. However, for the purpose of clarity and coherence, the current order of presentation is preferred. The researchers were careful to avoid any erroneous statements about the causal or temporal relationship between the two experiments.

3 The logistic models in this paper were estimated using a binomial logit-link function, where the intercept is presented on the log-odds scale. A value of 0 on this scale corresponds to a predicted probability of 0.50. Log odd values can be converted to probabilities using the following formula $p = \frac{{e^{x} }}{{1 + e^{x} }}$ where x is the log odd value of interest.
